# Lysine-specific demethylase KDM3A regulates ovarian cancer stemness and chemoresistance

**DOI:** 10.1038/onc.2016.320

**Published:** 2016-10-03

**Authors:** S Ramadoss, S Sen, I Ramachandran, S Roy, G Chaudhuri, R Farias-Eisner

**Affiliations:** 1 Department of Obstetrics and Gynecology, David Geffen School of Medicine at University of California, Los Angeles, CA, USA; 2 Department of Endocrinology, Dr ALM PG Institute of Basic Medical Sciences, University of Madras, Taramani Campus, Chennai 600 113, Tamil Nadu, India; 3 Molecular and Medical Pharmacology, David Geffen School of Medicine at University of California, Los Angeles, CA, USA

## Abstract

Ovarian cancer is the leading cause of death among all gynecological malignancies due to the development of acquired chemoresistance and disease relapse. Although the role of cancer stem cells (CSCs), a subset of tumor cells with the self-renewal and differentiation capabilities, in therapeutic resistance is beginning to be better understood, the significance of epigenetic regulatory mechanisms responsible for integrating the stemness with drug resistance remain poorly understood. Here we identified that lysine demethylase KDM3A as a critical regulator of ovarian cancer stemness and cisplatin resistance by inducing the expressions of pluripotent molecules Sox2 and Nanog and anti-apoptotic B-cell lymphoma 2 (Bcl-2), respectively. In addition, KDM3A induces ovarian cancer growth while antagonizing cellular senescence by repressing the expression of cyclin-dependent kinase inhibitor, p21^Waf1/Cip1^. The underlying mechanism of the noted biological processes include KDM3A-mediated stimulation of Sox2 expression, and demethylating p53 protein and consequently, modulating its target genes such as Bcl-2 and p21^Waf1/Cip1^ expression. Consistently, KDM3A depletion inhibited the growth of subcutaneously implanted cisplatin-resistant human ovarian cancer cells in athymic nude mice. Moreover, KDM3A is abundantly expressed and positively correlated with Sox2 expression in human ovarian cancer tissues. In brief, our findings reveal a novel mechanism by which KDM3A promotes ovarian CSCs, proliferation and chemoresistance and thus, highlights the significance of KDM3A as a novel therapeutic target for resistant ovarian cancer.

## Introduction

Ovarian cancer is a devastating disease and leading cause of death among all gynecological malignancies.^1^ American cancer society estimated that 22 280 women will be diagnosed with ovarian cancer and 14 240 women will die from the disease in the year 2016.^2^ Despite significant progress has been made over the past three decades, the overall 5-year survival rate of ovarian cancer patients remain <40% because majority of the cases are diagnosed with advanced stage disease.^3^ Even though surgery followed by platinum/taxane-based chemotherapy initially benefits the patients, disease relapse limits the survival rate as the recurrent tumor poorly responds to chemotherapy.^4^ Emerging evidences indicated that cancer stem cells (CSCs), a subpopulation of tumor cells with stem cell properties and molecular signature, are the underlying cause of recurrent tumor growth because these cells are reprogrammed to overcome the chemotherapy induced growth arrest and apoptosis.^5, 6^ Therefore, unveiling the mechanisms that promote ovarian CSCs and chemoresistance would turn out to be essential for devising a novel therapeutic strategy to target the chemotherapy resistant ovarian cancer.

Emerging consensus on the co-existence of stemness and therapeutic resistance in cancer driven the concerted efforts to identify the signaling and/or epigenetic mechanisms that control and or integrate these processes in cancer. For instance, tumor suppressor protein p53, which promotes cellular senescence and apoptosis has been identified to inhibit pluripotency.^7^ Indeed, in ovarian cancer, loss of p53 expression or function due to genetic mutation or post-translational modifications is often associated with hyper-proliferation, apoptosis resistance and stemness.^8, 9^ To date, numerous post-translational modifications including phosphorylation, acetylation, methylation and ubiquitylation have been reported to modulate p53 functions in tumorigenesis.^10^

Besides the genetic mutation, cancer progression is also controlled by epigenetic changes that reprogram the gene expression through DNA methylation and chromatin modifications.^11^ In recent years, numerous studies pointed out that epigenetic histone modification by lysine methyltransferases (KMTs) and demethylases (KDMs) have a crucial role in determining gene expression.^12, 13^ Importantly, a large group of Jumonji-C domain containing KDMs were discovered for their potential role in controlling gene expression during embryonic development, stem cell self-renewal and differentiation, genome integrity and oncogenesis.^14, 15^ Therefore, to identify the epigenetic changes that contribute to the ovarian cancer stemness and development of chemoresistance, we screened the expression of 24 Jumonji-C domain containing histone demethylases in parental and cisplatin-resistant ovarian cancer cells. We identified that lysine demethylase KDM3A/JMJD1A, which specifically demethylates H3K9me2, is highly expressed in platinum-resistant ovarian cancer cells. Knockdown of KDM3A induced cell cycle arrest, promoted cellular senescence and apoptosis in platinum-resistant ovarian cancer cells. Interestingly, we found that KDM3A employs dual mechanisms to control ovarian cancer by demethylating histone (H3K9me2) and non-histone protein, p53. Consistently, KDM3A depletion inhibited *in vivo* growth of ovarian cancer xenograft in mice and abundantly expressed in human ovarian cancer tissues.

## Results

### KDM3A is highly expressed in cisplatin-resistant ovarian cancer cells

**Figure 1:**
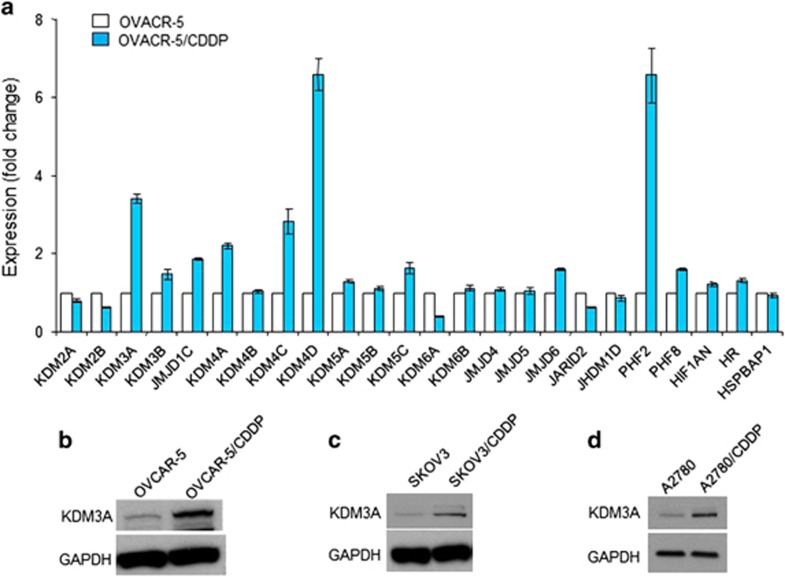
KDM3A is highly expressed in cisplatin-resistant ovarian cancer cells. (**a**) Quantitative RT-PCR analysis of histone demethylase family proteins mRNA expression in OVCAR-5 and OVCAR-5/CDDP cells. Each bar represents mean±s.d. of triplicate experiments. (**b**–**d**) Immunoblot analysis of KDM3A protein expression in parental and cisplatin-resistant OVCAR-5 (**b**), SKOV3 (**c**) and A2780 (**d**) cells.

To explore the epigenetic mechanisms of cisplatin resistance in ovarian cancer, we screened the expression of JMJD family histone demethylases in parental (OVCAR-5) and cisplatin-resistant (OVCAR-5/CDDP) ovarian cancer cell lines by real-time reverse transcriptase (RT)-PCR. As shown in Figure 1a, our screening identified that KDM3A, KDM4D and PHF2 were highly expressed in OVCAR-5/CDDP cells as compared with OVCAR-5 cells. Emerging evidences suggested that KDM3A is a hypoxic target gene and mediates the hypoxia-inducible factor-1α-induced tumor progression through epigenetic mechanisms.^16^ Since it is well established that hypoxic tumor microenvironment drives the aggressiveness and chemoresistance in ovarian cancer,^17, 18^ we focused our further studies on KDM3A. Next, we examined the protein expression of KDM3A in parental and cisplatin-resistant OVCAR-5 cells by immunoblotting. Consistent with the mRNA expression, OVCAR-5/CDDP cells highly expressed KDM3A protein as compared with parental cells (Figure 1b). Further, to check whether the high abundance of KDM3A correlated with platinum resistance in other ovarian cancer cells, we determined the KDM3A protein expression in parental and platinum-resistant SKOV3 and A2780 cells. Notably, all the cisplatin-resistant ovarian cancer cells consistently overexpressed KDM3A protein as compared with parental cell line (Figures 1b and d). Altogether, these results indicated that KDM3A might be a crucial epigenetic factor required for platinum resistance in ovarian cancer.

### KDM3A depletion inhibited cell cycle progression by inducing G_2_/M arrest

**Figure 2:**
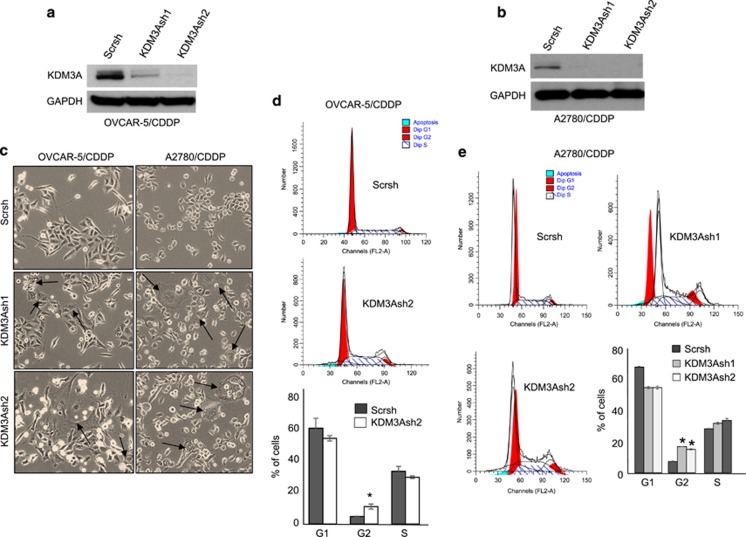
KDM3A knockdown induces cell cycle arrest in cisplatin-resistant ovarian cancer cells. (**a** and **b**) Immunoblot analysis of KDM3A expression in OVCAR-5/CDDP (**a**) and A2780/CDDP (**b**) cells expressing Scr and KDM3A shRNAs. (**c**) Photomicrograph showing the morphological changes in OVCAR-5/CDDP and A2780/CDDP cells following KDM3A knockdown. The giant vacuolated cells are indicated by arrows. (**d**) Cell cycle profile of OVCAR-5/CDDP cells expressing Scr and KDM3AshRNA as determined by flow cytometry. (**e**) Cell cycle profile of A2780/CDDP cells expressing Scr and KDM3A shRNAs as determined by flow cytometry. Each bar represents mean±s.d. of triplicate samples from representative experiments. *P<0.05, Student's *t*-test.

**Figure 3:**
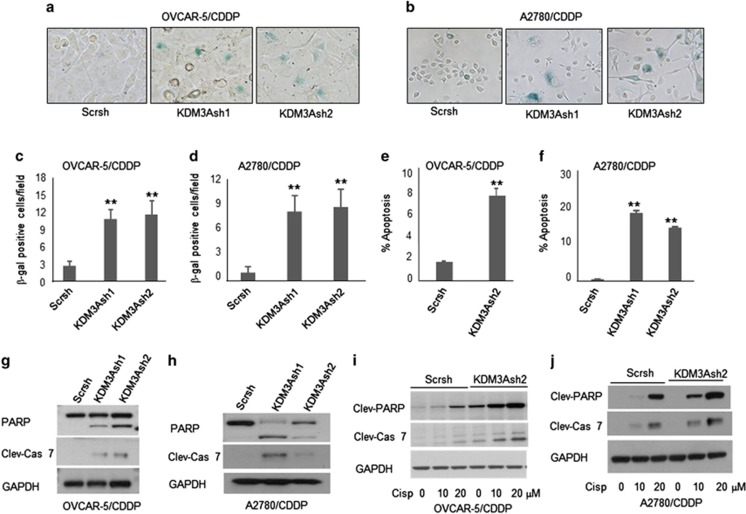
KDM3A depletion promotes cellular senescence and apoptosis in cisplatin-resistant ovarian cancer cells. (**a** and **b**) Senescence associated β-galactosidase staining of OVCAR-5/CDDP (**a**) and A2780/CDDP (**b**) cells expressing Scr and KDM3A shRNAs. (**c** and **d**) Quantification of β-galactosidase positive cells in OVCAR-5/CDDP (**c**) and A2780/CDDP (**d**) cells expressing Scr and KDM3A shRNAs. Each bar represents mean±s.d. of nine random fields counted from triplicate wells. **P<0.01, Student's *t*-test. (**e** and **f**) Quantification of apoptotic cells in Scr and KDM3A shRNAs expressing OVCAR-5/CDDP (**e**) and A2780/CDDP (**f**) cells. Each bar represents mean±s.d. of triplicate samples from representative experiments. **P<0.01, Student's *t*-test. (**g** and **h**) Immunoblot analysis of cleaved PARP and –caspase-7 expression in scrambled control and KDM3A-depleted OVCAR-5/CDDP (**g**) and A2780/CDDP (**h**) cells. (**i** and **j**) Immunoblot analysis of cleaved PARP and –caspase-7 expression in scrambled control and KDM3A knockdown OVCAR-5/CDDP (**i**) and A2780/CDDP (**j**) cells exposed to cisplatin.

To understand the functional role of KDM3A in resistant ovarian cancer, we stably knocked down KDM3A by lentiviral method in OVCAR-5/CDDP and A2780/CDDP cells by using two shRNAs targeting different sequences on KDM3A mRNA. As demonstrated by immunoblot, both the KDM3A shRNAs efficiently depleted KDM3A protein in OVCAR-5/CDDP and A2780/CDDP cells (Figures 2a and b). Surprisingly, we observed that significant proportion of KDM3A-depleted OVCAR-5/CDDP and A2780/CDDP cells became large, flat and vacuolated (Figure 2c). Moreover, our microscopic observation also revealed that KDM3A-depleted OVCAR-5/CDDP and A2780/CDDP cells grew relatively slower than scrambled control cells with high abundance of floating cell population. Taken together, these observations suggest that KDM3A might play a key role in ovarian cancer growth and survival. To check this possibility, we evaluated the cell cycle profile of OVCAR-5/CDDP and A2780/CDDP cells expressing scrambled and KDM3A shRNAs by flow cytometry. Interestingly, KDM3A depletion led to G_2_/M cell cycle arrest, which was accompanied by increased apoptosis (Figures 2d and e, 3e and f), indicating that KDM3A is crucial for ovarian cancer cell proliferation and survival.

### KDM3A promotes ovarian cancer growth and survival by inhibiting cellular senescence and apoptosis

Cellular senescence, a state of irreversible growth arrest, which plays a major role in aging and cancer is often accompanied by morphological changes such as cells becoming large, flat and multinucleated.^19^ Since we observed that growth arrest was accompanied by the presence of large, flat and vacuolated cells in KDM3A knockdown cells, we stained for senescence-associated β-galactosidase activity, a hallmark of cellular senescence. Indeed, both OVCAR-5/CDDP and A2780/CDDP cells expressing KDM3A shRNAs showed high abundance of senescence-associated β-galactosidase positive cells as compared with scrambled control cells (Figures 3a and d), indicating that KDM3A prevents the cellular senescence to promote sustained growth of ovarian cancer. Because we noticed a high proportion of floating cell population in KDM3A-depleted wells, we quantified the percentage of apoptotic cells by flow cytometry. As expected, KDM3A knockdown significantly increased the apoptotic cell death in OVCAR-5/CDDP and A2780/CDDP cells (Figures 3e and f). Further, to confirm the KDM3A shRNAs-induced apoptosis, we also examined the expressions of classical apoptotic markers such as cleaved PARP and caspase-7 by immunoblotting. In line, KDM3A knockdown induced the cleavage of caspase-7 and PARP in OVCAR-5/CDDP and A2780/CDDP cells (Figures 3g and h). These results clearly indicated that KDM3A knockdown itself is sufficient to induce apoptosis in platinum-resistant ovarian cancer cells. Next, we asked whether KDM3A depletion reverse the sensitivity of platinum-resistant cell lines to cisplatin treatment. To explore this, we exposed the scrambled control and KDM3A knockdown cells to cisplatin (10 and 20 μm) for 24 h and checked the expressions of apoptotic markers by western blot. Indeed, cisplatin treatment robustly induced the expression of cleaved PARP and caspase-7 in KDM3A knockdown OVCAR-5/CDDP and A2780/CDDP cells as compared with scrambled control cells (Figures 3i and j). Taken together, these results clearly indicated that KDM3A is required for the cell growth and confers chemoresistance in ovarian cancer.

### KDM3A controls ovarian cancer growth and chemoresistance by modulating p53-mediated expressions of p21 and Bcl-2

**Figure 4:**
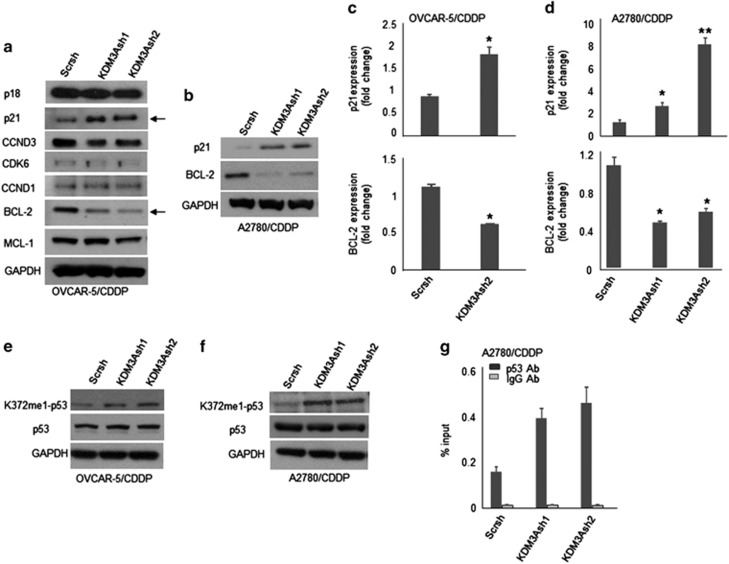
KDM3A knockdown inhibits Bcl-2 and induces p21 expression through p53 demethylation. (**a**) Immunoblot analysis of cell cycle and apoptotic regulatory protein expression in OVCAR-5/CDDP cells expressing Scr and KDM3A shRNAs. Changes in p21 and Bcl-2 expressions were indicated by arrows. (**b**) Immunoblotting of p21 and Bcl-2 expressions in A2780/CDDP cells expressing Scr and KDM3A shRNAs. (**c** and **d**) Real-time RT-PCR of p21 and Bcl-2 expression in OVCAR-5/CDDP (**c**) and A2780/CDDP (**d**) cells expressing Scr and KDM3AshRNAs. Each bar represents mean ± s.d. of triplicate samples from representative experiments. *P<0.05, Student's *t-* test. (**e** and **f**) Immunoblot analysis of p53-K372-me1 and p53 expressions in scrambled control and KDM3A shRNAs transfected OVCAR-5/CDDP (**e**) and A2780/CDDP (**f**) cells. (**g**) ChIP assay indicating p53 binding to p21 promoter in scrambled control and KDM3A shRNAs transduced A2780/CDDP cells. Each bar represents mean±s.d. of triplicate samples from representative experiments.

To gain the molecular insight into the mechanism by which KDM3A regulates ovarian cancer growth and chemoresistance, we screened the expression of key cell cycle and apoptotic regulatory proteins in scrambled control and KDM3A knockdown OVCAR-5/CDDP cells by immunoblot. Interestingly, KDM3A knockdown induced the cyclin-dependent kinase inhibitor 1, p21^Waf1/Cip1^, and reduced the anti-apoptotic B-cell lymphoma 2 (Bcl-2) expressions in OVCAR-5/CDDP cells (Figure 4a). Consistently, KDM3A depletion in A2780/CDDP cells was also associated with robust induction of p21 expression with concomitant decrease in Bcl-2 levels (Figure 4b). Next, to examine whether KDM3AshRNA-induced changes in p21 and Bcl-2 expressions are regulated at transcriptional or translational level, we quantified the mRNA expression by real-time RT-PCR. In line with immunoblot, cells expressing KDM3A shRNAs showed high abundance of p21 with reduced levels of Bcl-2 mRNA (Figures 4c and d), indicating that KDM3A regulates these genes at transcriptional level. The tumor suppressor protein p53 is a well-known regulator of p21 and Bcl-2 expressions.^20, 21^ The transcriptional activity of p53 can be regulated by post-translational modifications such as phosphorylation, acetylation and methylation.^10, 22^ Recent study showed that KDM3A inhibits p53 transcriptional activity by demethylating p53-K372me1 in breast cancer cells to represses apoptotic gene expression.^23^ Therefore, we hypothesized that KDM3A may influence p21 and Bcl-2 expressions by demethylating p53-K372me1 in ovarian cancer. To check this possibility, we assessed the expression of p53-K372me1 in scrambled control and KDM3A-depleted OVCAR-5/CDDP and A2780/CDDP cells by immunoblot. Indeed, KDM3A shRNAs consistently upregulated p53-K372me1 protein expression in OVCAR-5/CDDP and A2780/CDDP cells (Figures 4e and f). Previous report suggested that mono methylation of p53 at K372 enhances the transcriptional activity by stabilizing the chromatin bound p53.^24^ Therefore, we quantified the p53 occupancy on p21 promoter by chromatin immunoprecipitation (ChIP) assay. Consistently, anti-p53 antibody-enriched chromatin showed increased binding of p53 protein on p21 promoter in KDM3A-depleted cells (Figure 4g). Normal immunoglobulin G (IgG)-enriched chromatin was used as negative control.

### KDM3A promotes ovarian cancer stemness by epigenetically activating Sox2 expression

**Figure 5:**
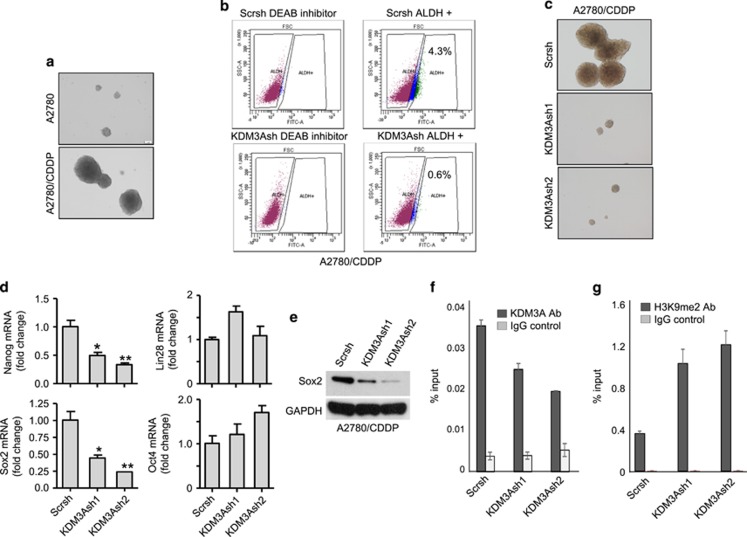
KDM3A controls ovarian CSCs by regulating Sox2 expression. (**a**) Representative image of tumorsphere-formation assay indicating high abundance of CSCs in A2780/CDDP cells. (**b**) Flow cytometry analysis of the abundance of CSCs in A2780/CDDP cells expressing Scr and KDM3AshRNA2. (**c**) Tumorsphere-formation assay of Scr and KDM3A shRNAs expressing A2780/CDDP cells. (**d**) Real-time RT-PCR of pluripotent markers expression in Scr and KDM3A shRNAs transfected A2780/CDDP cells. Each bar represents mean±s.d. of triplicate samples from representative experiments. *P<0.05, **P<0.01 Student's *t*-test. (**e**) Immunoblot analysis of Sox2 expression in A2780/CDDP cells expressing Scr and KDM3A shRNAs. (**f** and **g**) ChIP assays indicating KDM3A (**f**) and H3K9me2 (**g**) localization on Sox2 promoter in A2780/CDDP cells expressing Scr and KDM3A shRNAs. Each value is mean±s.d. of triplicate samples from a representative experiment.

CSC hypothesis states that subset of tumor cells with self-renewal and differentiation potential initiate tumor development and progression.^25^ These long-lived CSCs are the underlying cause of disease relapse and treatment failure in ovarian cancer because these cells are endowed with apoptosis resistance.^26^ In light of the emerging consensus on CSCs hypothesis, we attempted to investigate whether platinum resistance co-exist with stemness in ovarian cancer by utilizing A2780/CDDP cells. We conducted tumorsphere assay, which is an indicator of CSCs, using parental and cisplatin-resistant A2780 cells. Indeed, A2780/CDDP cells formed more and larger tumorsphere in suspension culture as compared to parental cells (Figure 5a). Because our findings indicated that high abundance of KDM3A coincide with cancer stemness and chemoresistance in ovarian cancer, we hypothesized that KDM3A might be a critical regulator of CSCs in ovarian cancer. To check this possibility, we determined the percentage of aldehyde dehydrogenase 1 (ALDH-1)-positive cells, a hallmark of stem cells, between A2780/CDDP cells expressing scrambled and KDM3AshRNA2 by fluorescence-activated cell sorting. Interestingly, KDM3A knockdown significantly reduced the ALDH-positive cells from 4.3 to 0.6% in A2780/CDDP cells (Figure 5b). Consistently, KDM3A depletion also inhibited the ability of A2780/CDDP cells to form and propagate tumorsphere in suspension culture (Figure 5c). Altogether, these results indicated that KDM3A is required to maintain CSCs population in ovarian cancer. Next, to identify the molecular mechanism of KDM3A-mediated ovarian cancer stemness, we assessed the expression of stem cell pluripotent markers such as Sox2, Oct4, Nanog and Lin28 by real-time RT-PCR. Intriguingly, while KDM3A knockdown significantly inhibited the Sox2 and Nanog expressions in A2780/CDDP cells, Oct4 and Lin28 expressions were weakly upregulated (Figure 4d). Next we checked the Sox2 and Nanog protein expression by western blot. Consistent with real-time RT-PCR, immunoblotting also revealed that KDM3A knockdown inhibited Sox2 expression in A2780/CDDP cells (Figure 5e). Despite the repeated attempt, we were unable to detect Nanog protein expression by immunoblot in A2780/CDDP cells. The real-time RT-PCR also revealed that the cycle threshold (Ct) value for Nanog was too high, indicating that Nanog might be weakly expressed in A2780/CDDP cells. Recent study indicated that chromatin modification through H3K9 methylation regulates Sox2 expression in lung cancer.^27^ Because KDM3A is known to activate gene expression through H3K9me2 demethylation,^28, 29, 30^ we checked the binding of KDM3A and the level of its substrate, H3K9me2 on Sox2 promoter by ChIP assay. Significant amount of KDM3A protein was detected on Sox2 promoter and KDM3A knockdown reduced KDM3A occupancy with concomitant increase in H3K9me2 (Figures 5f and g), indicating that KDM3A may epigenetically activates Sox2 expression to promote ovarian cancer stemness.

### KDM3A is required for *in vivo* tumor growth and abundantly expressed in human ovarian cancer tissues

**Figure 6:**
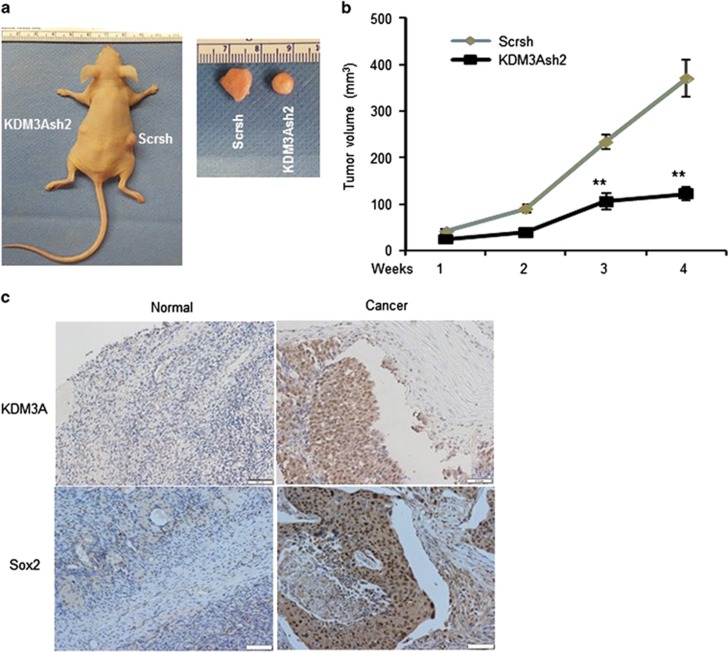
KDM3A is required for *in vivo* tumor growth and highly expressed in human ovarian cancer tissues. (**a**) Representative photograph of mice bearing the tumors formed by Scr and KDM3AshRNA2 transfected A2780/CDDP cells. (**b**) Tumor growth curve of subcutaneously injected Scr and KDM3AshRNA2 expressing A2780/CDDP cells in nude mice. Data are mean±s.d., *n=*5. **P<0.01, Student's *t-*test. (**c**) Representative images of KDM3A and Sox2 expressions in human ovarian cancer and adjacent normal tissues.

The results discussed so far clearly showed that KDM3A is crucial for tumor cell proliferation, apoptosis resistance and CSCs maintenance. Therefore, we next sought to determine the importance of KDM3A for *in vivo* growth of human ovarian cancer by utilizing the mouse tumor xenograft model. We subcutaneously inoculated A2780/CDDP cells expressing scrambled and KDM3AshRNA2 on the right and left flanks of nude mice, respectively. Once the palpable tumors were formed, we periodically measured the tumor growth and calculated the tumor volume. Indeed, KDM3A depletion significantly inhibited the ovarian cancer growth *in vivo* (Figures 6a and b). To further determine whether KDM3A epigenetically activates Sox2 expression to control ovarian cancer progression, we determined KDM3A and Sox2 protein expression in human ovarian cancer tissue array containing primary and metastatic ovarian cancer and adjacent normal tissues. KDM3A and Sox2 expressions were significantly elevated in ovarian cancer than the adjacent normal tissues (Figure 5c and Table 1). Moreover, the abundance of KDM3A was positively correlated with Sox2 levels (Table 2), indicating the functional significance of KDM3A in human ovarian cancer.

## Discussion

Existence of strong association between chemoresistance and disease relapse in ovarian cancer underscores the need to identify the molecular basis of resistant phenotype to develop targeted therapy. The findings presented here demonstrated for the first time that histone demethylase, KDM3A is crucial for the ovarian cancer cells to successfully progress through the critical stages of tumor progression such as cell proliferation, maintenance of CSCs and development of chemoresistance. To control these processes, KDM3A employs two distinct mechanisms; one by demethylating histone (H3K9me2) and the other by targeting a non-histone protein, p53. Mechanistically, while activating Sox2 expression by erasing the repressive methylation (H3K9me2) mark, KDM3A modulates p21 and Bcl-2 expression possibly through p53-K372me1 demethylation. The dual mechanisms we reported here are in consistent with the recent findings on breast cancer, where KDM3A induced pro-invasive genes and repressed pro-apoptotic genes by demethylating histone (H3K9me2) and non-histone protein p53, respectively.^23^ Even though KDM3A is mechanistically engaged in similar pathways, the target genes and cellular functions controlled by KDM3A vary between breast and ovarian cancer. Especially, KDM3A loss in ovarian cancer induces replicative senescence and cell cycle arrest but no such effects were seen in breast cancer cells. However, KDM3A renders chemoresistance in both the cancers by modulating p53 target gene expressions. In view of our finding, we attempted to analyze the patient data sets from oncomine database to identify the correlation between KDM3A expression and cisplatin resistance in ovarian cancer. Our mining indicated that no such data sets are available in the online database. However, two different studies^31, 32^ indicated that KDM3A mRNA expression was high in ovarian serous cystadenocarcinoma than the normal ovarian epithelium (supplementary Figure 1a and b).

Hypoxic tumor microenvironment drives ovarian cancer aggression by promoting the cancer stemness, cellular growth, metastasis and therapeutic resistance.^33^ The hypoxia-driven aggressiveness is mainly dependent on the stabilization and transactivation of the transcription factor, hypoxia-inducible factor-1α.^34^ Recently, KDM3A was identified as a potential target of hypoxia-inducible factor-1α and mediates hypoxia-induced gene expression to control tumor growth.^16^ Since our study indicated KDM3A as ovarian cancer oncogene and is required for tumor growth and apoptosis resistance, it is possible that KDM3A might be critical mediator of hypoxia-induced tumor aggressiveness and thus could be a potential target to inhibit hypoxia-driven ovarian cancer.

Compelling evidences suggest that tumor initiating CSCs are responsible for the development and progression of various cancers.^35^ Because CSCs are endowed with high metastatic and apoptosis-resistant potential, chemotherapy often failed to eliminate CSC population. Consequently, surviving CSCs initiate recurrent tumor growth, promote metastasis and causes treatment failure. Therefore, in addition to tumor bulk, targeting CSCs with specific inhibitors would improve the survival rate and clinical outcome in ovarian cancer patients. Hence, recent research efforts are intensified toward identifying the genetic and epigenetic changes that confers chemoresistance to CSCs. In this regard, our results indicating KDM3A as a key epigenetic factor controlling ovarian CSCs may offer a new perspective to develop an inhibitor that specifically target KDM3A to eliminate CSC population and overcome chemoresistance in ovarian cancer. Since the demethylating activity and biological functions of KDM3A resides in the catalytic subunit, it can be a potential druggable target for small molecules that specifically target the rigid catalytic domain and thus can be exploited to device a novel therapy to target-resistant ovarian cancer.

## Materials and methods

### Cell lines and generation of stable cells

Parental (OVCAR-5, SKOV3 and A2780) and cisplatin-resistant (OVCAR-5/CDDP, SKOV3/CDDP and A2780/CDDP) cells were cultured in RPMI-1640 medium containing 10% heat-inactivated fetal bovine serum and antibiotics (streptomycin and penicillin) at 37 °C in 5% CO_2_ and 95% air. Parental and cisplatin-resistant cell lines were generously provided by Dr Oliver Dorigo, Stanford University Medical Center, Stanford, CA, USA. To stably knockdown KDM3A in platinum-resistant ovarian cancer, lentiviruses expressing human KDM3A shRNAs (TRCN0000021150 and TRCN0000021152, Dharmacon, Lafayette, CO, USA) were packaged and generated in 293 T cells as described elsewhere.^36, 37^ The cells were seeded in 10 cm plates overnight and then infected with lentiviral particles. After 24 h, cells were selected with puromycin (1 μg/ml).

### Cell cycle analysis

Cisplatin-resistant ovarian cancer cells stably expressing scrambled or KDM3A shRNAs were harvested with floating cells and centrifuged at 1000 rpm for 5 min. The supernatant was discarded, and the cells were washed and re-suspended in phosphate-buffered saline. Single-cell suspension was fixed in ice-cold 70% ethanol for 2 h at 4 °C. The cells were centrifuged to remove the ethanol and washed twice with phosphate-buffered saline. The fixed cells were suspended in phosphate-buffered saline containing 1 mg/ml propidium iodide, 0.1% Triton X-100 and 2 μg DNase-free RNase and incubated for 30 min at room temperature in dark. Flow cytometry was done with a FACSCalibur analyzer (BD Biosciences, San Jose, CA, USA), capturing 10 000 events for each sample.

### Quantification of cellular senescence by β-galactosidase staining

Scrambled control and KDM3A-depleted OVCAR-5/CDDP and A2780/CDDP cells were plated onto six-well culture plates overnight. The senescence assay was performed using senescence β-galactosidase staining kit as per manufacturer's instructions (Cell Signaling Technology, Beverly, MA, USA).

### RNA extraction and real-time RT-PCR

Total RNA was extracted with miRNeasy kit according to the manufacturer's instruction (Qiagen, Valencia, CA, USA). Two microgram of total RNA was reverse transcribed into cDNA using iScript reverse transcription kit (Bio-Rad, Hercules, CA, USA). Real-time RT-PCR analysis was carried out using iQ SYBR Green Supermix (Bio-Rad) on an iCycler iQ real-time PCR detection system (Bio-Rad). Primers used for quantitative RT-PCR were listed in the supplementary methods (Supplementary Table 1).

### Western blot analysis

Cells grown in 10 cm culture plates were collected using the cell scraper and centrifuged at 1500 rpm for 5 min. The supernatants were carefully removed and the cell pellets were lysed using 200 μl of radioimmunoprecipitation assay buffer for 30 min on ice. The cell lysates were centrifuged at 10 600 *g* at 4 °C, the supernatants were collected and stored at −80 °C until used. Equal amount of proteins (25–50 μg) were resolved on sodium dodecyl sulfate polyacrylamide gel electrophoresis (SDS-PAGE) and transferred onto Polyvinylidene difluoride (PVDF) membrance using XCell SureLock Blot Module (Invitrogen, Carlsbad, CA, USA). The blot transferred membranes were blocked for 1 h in 5% milk and incubated with primary antibodies overnight. After washing, the membranes were incubated with horseradish peroxidase–conjugated anti-rabbit or anti-mouse IgG (Santa Cruz, Dallas, Texas, USA) for 1 h. The immunocomplexes on the membrane were detected using enhanced chemiluminescence reagents (GE Healthcare Biosciences, Pittsburgh, PA, USA). List of primary antibodies and their dilutions were indicated in supplementary methods (Supplementary Table 3).

### ChIP assay

ChIP assays were performed using a ChIP assay kit following the manufacturer's protocol (Upstate Biotechnology, Lake Placid, NY, USA). Cells (2 × 10^6^) were pre-incubated with a dimethyl 3,3′-dithiobispropionimidate-HCl (Pierce Biotechnology, Waltham, MA, USA) solution (5 mmol) for 30 min on ice and then treated with formaldehyde. The ChIP-enriched DNA samples were quantified by real-time PCR and the data are expressed as a percentage of input. The primer pairs used for ChIP assays are described in the supplementary methods (Supplementary Table 2).

### Quantification of ALDH-positive cells

The ALDEFLUOR kit was used to determine the percentage of cells with high ALDH activity following the manufacturer's protocol (STEMCELL Technologies Inc., Vancouver, BC, Canada). In brief, trypsinized cells were washed with phosphate-buffered saline and re-suspended in ALDEFLUOR assay buffer (2 × 10^5^ cells/ml) and incubated with ALDEFLUOR substrate with or without ALDH inhibitor, diethylaminobenzaldehyde (DEAB) for 30 min at 37 °C in water bath. Fluorescence-activated cell sorting method was used to analyze and determine percentage of ALDH-positive cells in the samples.

### Tumorsphere-formation assay

Cells were trypsinized and seeded (about 4000 cells/well) onto ultra-low attachment six-well culture plate containing 2.5 ml of sphere forming media (Dulbecco's Modified Eagle Medium/F12 50:50 containing 1% supplement B, 20 ng/ml EGF and 10 ng/ml fibroblast growth factor) and cultured for 14 days. The tumorspheres were photographed using the microscope.

### *In vivo* tumor growth assay

All the experiments using animals were performed in accordance with protocol approved by the UCLA Committee on Animal Care. For tumor xenograft studies, 6–8 weeks athymic nude (female) mice were used. A2780/CDDP cells expressing scrambled and KDM3AshRNA2 (5 × 10^5^) were injected subcutaneously into the right and left flanks, respectively. Once the palpable tumors were noticed, tumor growth was determined periodically and tumor volume (mm^3^) was calculated.

### Immunohistochemical staining of human ovarian cancer tissue

Human ovarian cancer tissue array (Cat No BC110118) containing primary and metastatic ovarian cancer and adjacent normal tissues was purchased from US Biomax, Rockville, MD, USA. The tissue array slides were deposited to UCLA pathology core for immunohistochemical (IHC) staining. The intensity of immunostaining was scored as no (0), weak (+), moderate (++) and strong (+++). The Fisher's exact test was applied to test the differences in the staining intensity and the correlation between KDM3A and Sox2 expression in adjacent normal and ovarian cancer tissues.

### Oncomine data analysis

Microarray data sets from oncomine database was analyzed for KDM3A mRNA expression between normal and ovarian cancer.^31, 32^ The details of the methods and statistical calculations applied were described elsewhere.^36, 37^

### Statistical analysis

The data were subjected to statistical analyses using SAS STAT version 9.1 (SAS Institute Inc.,Cary, NC, USA). Independent means were compared using unpaired Student's *t*-tests.

## Figures and Tables

**Table 1:** KDM3A is highly expressed in human ovarian cancer tissues

	*KDM3A*				*Sox2*			
	*0*	*+*	*++*	*+++*	*0*	*+*	*++*	*+++*
Normal	11%	89%	0%	0%	33%	67%	0%	0%
	(1/9)	(8/9)	(0/9)	(0/9)	(3/9)	(6/9)	(0/9)	(0/9)
Cancer**	0%	8%	57%	35%	0%	8%	57%	35%
	(0/63)	(5/63)	(36/63)	(22/63)	(0/63)	(5/63)	(36/63)	(22/63)

Adjacent normal ovarian tissues (Normal; *n=*9) along with human ovarian cancer including metastatic tumor tissues (cancer; *n=*63) were stained for KDM3A and Sox2. The intensity of the staining was scored as negative (0), weak (+), moderate (++) and strong (+++). **P< 0.01 normal vs ovarian cancer.

**Table 2:** Expression of KDM3A correlates with the Sox2 levels in human ovarian cancer

	KDM3A**					
		*0*	*+*	*++*	*+++*	*Total*
Sox2	0	0	3	0	0	3
	+	1	7	3	0	11
	++	0	1	24	11	36
	+++	0	2	9	11	22
Total		1	13	33	25	72

Adjacent normal ovarian tissues (*n=*9) and ovarian cancer tissues (*n=*63) were stained for KDM3A and Sox2. The staining score calculations were detailed in Table 1. Fisher's exact test *P*=0.000016.
