# Erratum: Lysine-specific demethylase KDM3A regulates ovarian cancer stemness and chemoresistance

**DOI:** 10.1038/onc.2017.331

**Published:** 2017-09-18

**Authors:** S Ramadoss, S Sen, I Ramachandran, S Roy, G Chaudhuri, R Farias-Eisner

**Keywords:** Oxidoreductases, Cancer therapeutic resistance, Ovarian cancer, Cell signalling

**Correction to:** *Oncogene* (2017) **36,** 1537–1545; doi:10.1038/onc.2016.320; published online 3 October 2016

Following the publication of this article the authors note that they inadvertently omitted the correct acknowledgement for the support from the NIH and The Carl and Roberta Deutsch Foundation for the above referenced paper.

The corrected acknowledgement should read:

We acknowledge The Carl and Roberta Deutsch Foundation (PI: Farias-Eisner), Kelly Day, and NIH/NCI U54CA143930 Grant (PI: Farias-Eisner) for their financial support. We thank Professor Oliver Dorigo, Stanford University Medical Center, for the generous gift of cisplatin-resistant ovarian cancer cell lines. We thank Dr Xiangming Ding, UCLA Clinical Microarray Core, for the statistical assistance.

The authors apologise for any inconvenience caused by this error.

