# Supplementary figures and images for: Lysine-specific demethylase KDM3A regulates ovarian cancer stemness and chemoresistance
Source: Oncogene. 2016 Oct 3;36(11):1537–45. doi: 10.1038/onc.2016.320 (PMC5357761; doi:10.1038/onc.2016.320)

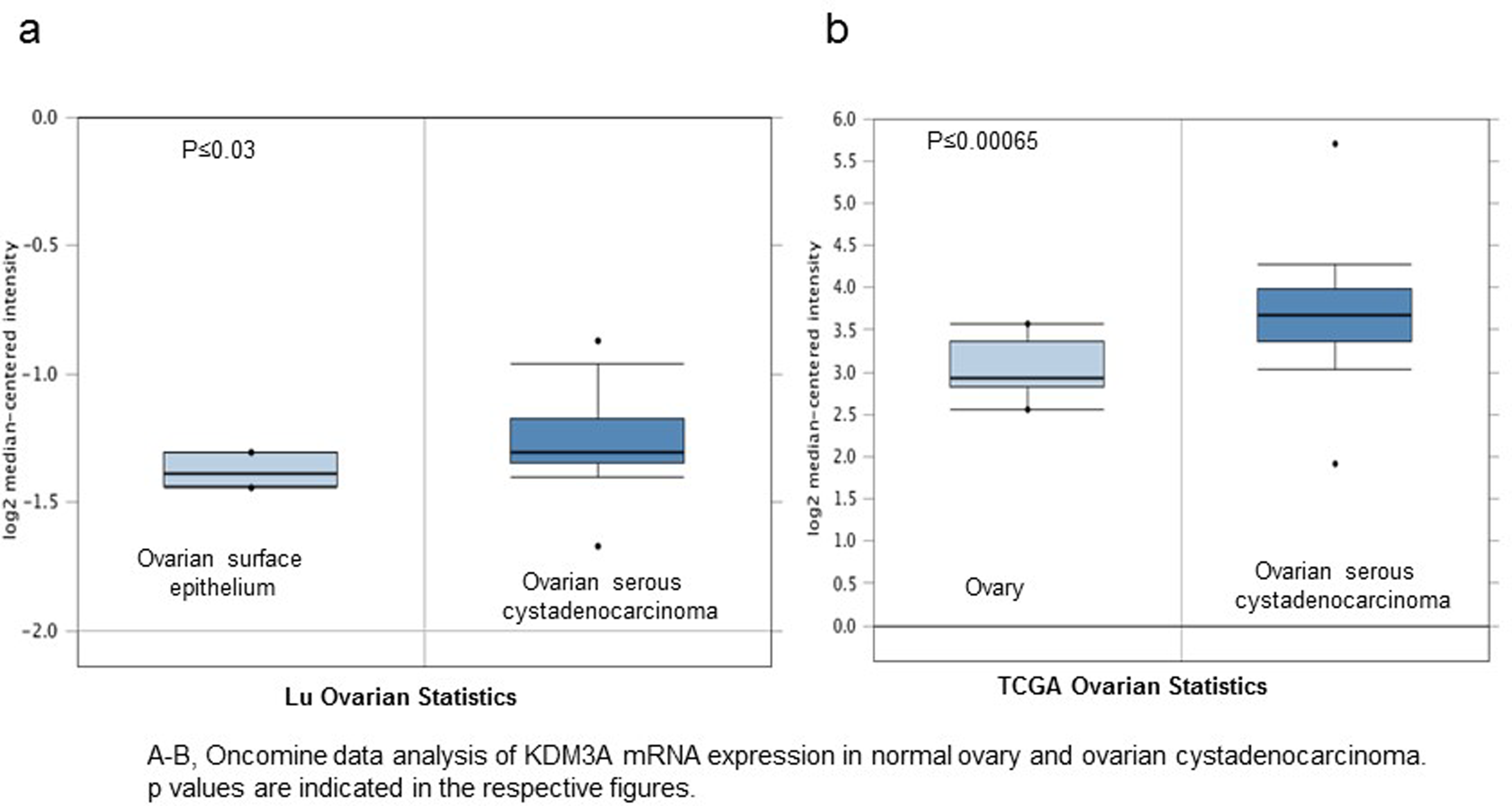

Supplement: Supplementary Figure [file onc2016320x1.tif]
