# Supplementary material for: Lysine-specific demethylase KDM3A regulates ovarian cancer stemness and chemoresistance
Source: Oncogene. 2016 Oct 3;36(11):1537–45. doi: 10.1038/onc.2016.320 (PMC5357761; doi:10.1038/onc.2016.320)
Supplement: Supplementary Information [file onc2016320x2.docx]

Supplementary methods related to:

**Lysine-specific demethylase KDM3A regulates ovarian cancer stemness and chemoresistance**

Sivakumar Ramadoss^1*^, Suvajit Sen^1^, Ilangovan Ramachandran^2^, Sohini Roy^1^, Gautam Chaudhuri^1,3^ and Robin Farias-Eisner^1*^

^1^Department of Obstetrics and Gynecology, David Geffen School of Medicine at University of California, Los Angeles, CA 90095, USA.

^2^Department of Endocrinology, Dr. ALM PG Institute of Basic Medical Sciences, University of Madras, Taramani Campus, Chennai - 600113, Tamil Nadu, India.

^3^Molecular and Medical Pharmacology, David Geffen School of Medicine at University of California, Los Angeles, CA 90095, USA.

Running Title: Oncogenic role of KDM3A in ovarian cancer

*To whom correspondence should be addressed

Dr. Sivakumar Ramadoss, PhD / Dr. Robin Farias-Eisner, MD, PhD,

Department of Obstetrics and Gynecology,

David Geffen School of Medicine at University of California,

Los Angeles, CA 90095, USA.

Telephone: +1-310-825-8022/ +1-310-206-8914

Fax: +1-310-206-3670 /+1-310-206-2057

Email: [ramasiva@ucla.edu](mailto:ramasiva@ucla.edu) / [rfeisner@mednet.ucla.edu](mailto:rfeisner@mednet.ucla.edu)

Table 1

Quantitative Real-time PCR primers (Most of the histone demethylase family primers were selected from previous publication by Zaho *et al*., 2013).

| KDM2A Forward | ATAACCAACCGTTCCCACCT |
| --- | --- |
| KDM2A Reverse | TGCCCAGTCCATCATAATCC |
|  |  |
| KDM2B Forward | CAGTGGGTGGAGGGACTAAA |
| KDM2B Reverse | ACTGAGGTGGAGCTTGGAGA |
|  |  |
| KDM3A Forward | ATGCCCACACAGATCATTCC |
| KDM3A Reverse | CTGCACCAAGAGTCGGTTTT |
|  |  |
| KDM3B Forward | AACTTCCTCAAACCCCCTTG |
| KDM3B Reverse | CCCATCACCATCTCCTTCAC |
|  |  |
| JMJD1C Forward | TCCAGAATCCCAGTCACCAC |
| JMJD1C Reverse | CAGCAAATCCCGTAAGGTTG |
|  |  |
| KDM4A Forward | CAGAGGACCAAGCCATTGAT |
| KDM4A Reverse | ATTGGCTGAACACCGAGAAC |
|  |  |
| KDM4B Forward | GGGGAGGAAGATGTGAGTGA |
| KDM4B Reverse | CTATGGGTGCCTCCTTCTCA |
|  |  |
| KDM4C Forward | TGCCTGAGGTTCTGTCCATT |
| KDM4C Reverse | GCTGCTATCTGGCTTGTGGT |
|  |  |
| KDM4D Forward | AAATATGTACGGGGCAACCA |
| KDM4D Reverse | TACTCAGACCTGGGGGTACG |
|  |  |
| KDM5A Forward | TGAACGATGGGAAGAAAAGG |
| KDM5A Reverse | AGCGTAATTGCTGCCACTCT |
|  |  |
| KDM5B Forward | TTGGGATTGAAAAGGAAGCA |
| KDM5B Reverse | CAGCAATTTCCCTTCATTGG |
|  |  |
| KDM5C Forward | CAGGGCTTACTGGAGAATGG |
| KDM5C Reverse | TTCTCATCCAGGGTCACCTC |
|  |  |
| KDM6A Forward | CGTGTCGTATCAGCAGGAAA |
| KDM6A Reverse | CACCCCAGTAACCTTCAGGA |
|  |  |
| KDM6B Forward | CTGATGCTAAGCGGTGGAAG |
| KDM6B Reverse | TGTTGATGTTGACGGAGCAG |
|  |  |
| JMJD4 Forward | ACTGGGTCAATGGCTTCAAC |
| JMJD4 Reverse | AGGACCAGGAGCCTCTTCTC |
|  |  |
| JMJD5 Forward | ACATCAGCATCCCCGACTAC |
| JMJD5 Reverse | AGGGTACAGAGCCCCTGACT |
|  |  |
| JMJD6 Forward | TATTTATCCCCGGACACAGC |
| JMJD6 Reverse | TACCGTCTTGTGCCATACCA |
|  |  |
| JHDM1D Forward | TATTCAGGGCATGCTGTCTATG |
| JHDM1D Reverse | GGGATCCTGGAGAGAGTTTCTT |
|  |  |
| JARID2 Forward | CTGTCTGGAGTGTGCTCTGC |
| JARID2 Reverse | ACGTCCACTGTCGCTCTCTT |
|  |  |
| PHF2-forward | ATCTTTAAGTCCCGGTCGAAG |
| PHF2-reverse | TTCCTCTTGGCACTCTTTT |
|  |  |
| PHF8 Forward | CTGATGATGATGACCCTGCTT |
| PHF8 Reverse | TTCTTCTTTTGGGCCTTCTGT |
|  |  |
| HIF1AN Forward | ACAATCCCGACTACGAGAGGT |
| HIF1AN Reverse | GCCACTTTCTGATGAGCTTTG |
|  |  |
| HR Forward | CAGTCAGCGTCACTCAGCA |
| HR Reverse | CGATCCCAGACACCTAGCA |
|  |  |
| HSPBAP1 Forward | AAGCTCAAAGACATGCGGTTA |
| HSPBAP1 Reverse | CAGGCTCTGGTATTTTGTGGA |
|  |  |
| p21 Forward | TGAGCCGCGACTGTGATG |
| p21 Reverse | GTCTCGGTGACAAAGTCGAAGTT |
|  |  |
| BCL-2 Forward | CTGCACCTGACGCCCTTCACC |
| BCL-2 Reverse | CACATGACCCCACCGAACTCAAAGA |
|  |  |
|  |  |
| Sox2 Forward | GGGAAATGGGAGGGGTGCAAAAGAGG |
| Sox2 Reverse | TTGCGTGAGTGTGGATGGGATTGGTG |
|  |  |
| Oct-4 Forward | GTGGAGGAAGCTGACAACAA |
| Oct-4 Reverse | ATTCTCCAGGTTGCCTCTCA |
|  |  |
| Nanog Forward | AGTCCCAAAGGCAAACAACCCACTTC |
| Nanog Reverse | TGCTGGAGGCTGAGGTATTTCTGTCTC |

Zhao W, Li Q, Ayers S, Gu Y, Shi Z, Zhu Q, et al. Jmjd3 inhibits reprogramming by upregulating expression of INK4a/Arf and targeting PHF20 for ubiquitination. Cell 2013;152:1037-50.

Table 2

ChIP primers

| p21 Forward | GTGGCTCTGATTGGCTTTCTG |
| --- | --- |
| p21 Reverse | CTGAAAACAGGCAGCCCAAG |
|  |  |
| Sox2 F | CTTAGACGAGGCTTTGTTTG |
| Sox2 R | GGGTTAGAGGAGGATGAGAT |

Table 3

List of Antibodies used

| **Antibody** | **Company** | **Cat Number** | **Dilution, Western/IHC** |
| --- | --- | --- | --- |
| KDM3A | Bethyl Laboratories | A301-539A | 1:1000/1:200 |
| PARP | Cell Signaling | 9532 | 1:2000 |
| Cleaved PARP | Cell Signaling | 5625 | 1:2000 |
| Cleaved-Cas-7 | Cell Signaling | 8438 | 1:2000 |
| p18 | Cell Signaling | 2896 | 1:2000 |
| p21 | Cell Signaling | 2947 | 1:2000 |
| CCND3 | Cell Signaling | 2936 | 1:2000 |
| CDK6 | Cell Signaling | 3136 | 1:2000 |
| CCND1 | Cell Signaling | 2978 | 1:2000 |
| BCL-2 | Santa Cruz | Sc-7382 | 1:500 |
| MCL-1 | Santa Cruz | Sc-819 | 1:500 |
| p53 ChIP Ab | Invitrogen | 49-1031 | 2 ug for ChIP |
| p53 | Cell Signaling | 2527 | 1:2000 |
| p53-K372me1 | Abcam | ab16033 | 1:500 |
| Sox2 | Cell Signaling | 3579 | 1:2000 |
| Sox2 IHC | Abcam | Ab97959 | 1:200 |
| Chip H3K9me2 | Millipore | CS200587 | 2 ug for ChIP |
| GAPDH | Santa Cruz | SC-47724 | 1:1000 |
